# 3′ UTR Deletion of *FBXO28* in a Patient with Brain Abnormalities and Developmental Delay

**DOI:** 10.3390/genes14091687

**Published:** 2023-08-25

**Authors:** Xin Bi, Maureen S. Mulhern, Erica Spiegel, Ronald J. Wapner, Brynn Levy, Jennifer M. Bain, Jun Liao

**Affiliations:** 1 Department of Pathology and Cell Biology, Columbia University Irving Medical Center, New York, NY 10032, USA; 2 Department of Neurology, Columbia University Irving Medical Center, New York, NY 10032, USA; 3 Department of Obstetrics and Gynecology, Columbia University Irving Medical Center, New York, NY 10032, USA; 4 Department of Pediatrics, Columbia University Irving Medical Center, New York, NY 10032, USA

**Keywords:** 3′ UTR deletion, brain abnormalities, chromosome 1q42.11 region, developmental delay, *FBXO28*

## Abstract

Constitutional deletions of chromosome 1q42 region are rare. The phenotype spectrum associated with this copy number change is variable, including developmental delay, intellectual disability, seizures, and dysmorphology. This study describes a patient with developmental delays and brain abnormalities. G-banded karyotype, FISH, SNP oligonucleotide microarray analysis (SOMA), and whole exome sequencing analysis were performed. Postnatal reanalysis of prenatal SOMA and follow-up parental testing revealed a paternally inherited 63 kb deletion at 1q42.11 in the patient. We characterized the clinical features of this patient, providing insight into the clinical phenotype associated with deletions of the 1q42.11 sub-band. Our study provides new evidence supporting the potential functional importance of the *FBXO28* 3′ UTR region and the hypothesis that *FBXO28* is a critical gene in the pathogenesis of chromosome 1q41q42 microdeletion syndrome. It also highlights the different goals and reporting criteria between prenatal and postnatal microarray tests.

## 1. Introduction

Copy number variation plays a crucial role in shaping genetic diversity in humans and can contribute to the manifestation of traits or be associated with complex diseases [1]. Constitutional interstitial deletions of chromosome 1q41q42 region are rare. Since 2007, a series of cases describing patients with deletions involving the chromosome 1q41q42 region has led to the clinically recognizable 1q41q42 microdeletion syndrome (OMIM#:612530). Common clinical features of patients with this syndrome include developmental delay, intellectual disability, dysmorphology, and a predisposition to seizures. Brain anomalies, as evaluated by imaging, are frequently seen in these individuals. Congenital anomalies such as diaphragmatic hernia, cleft palate, and cardiac defects are less often observed but have also been described [2]. Considerable interest in refining the critical region of the 1q41q42 microdeletion syndrome has implicated *FBXO28* as a candidate causative gene for the 1q41q42 microdeletion syndrome, particularly contributing to the neurodevelopmental and epileptic phenotypes [3,4,5]. 

The *FBXO28* gene encodes a member of the F-box protein family that forms a Skp-Cullin-F-Box (SCF) complex to act as a protein-ubiquitin ligase. More recent reports have suggested a role for *FBXO28* in causing monogenic disease [2,4]. Mutations in *FBXO28* are associated with developmental and epileptic encephalopathy-100 (OMIM:619777), a severe neurologic disorder characterized by global developmental delay and the onset of variable types of seizures in the first months or years of life. Affected individuals have been reported with missense, nonsense, and frameshift variants in *FBXO28*, with all patients presenting with seizures and the majority exhibiting intellectual disability and brain abnormalities [2,4]. 

The aim of this study was to identify the underlying cause of the neurodevelopmental phenotype in our 28-month-old female patient through molecular cytogenetic characterization. We identified a novel 63-kb deletion within chromosome 1q42, which encompasses *DEGS1* and the 3′ untranslated region (UTR) of *FBXO28*. This finding was made through the reanalysis of prenatally acquired SNP oligonucleotide microarray analysis (SOMA) results, highlighting the differences in reporting criteria and goals between prenatal and postnatal tests. Furthermore, our study provides new evidence supporting the hypothesis that *FBXO28* plays an important role in the pathogenesis of chromosome 1q41q42 microdeletion syndrome, and it also suggests the potential functional importance of the 3′ UTR region in *FBXO28*.

## 2. Methods and Results

### 2.1. Cytogenetic Analysis

G-banded chromosome analysis was performed on cultured cells from an amniotic fluid sample with a resolution of approximately 425 bands, according to standard protocols. Fluorescence In Situ Hybridization (FISH) analysis was performed with chromosome-specific Vysis probes (Abbott Molecular Inc., Des Plaines, IL, USA) in 50 uncultured cells from an amniotic fluid sample.

### 2.2. SNP Oligonucleotide Microarray Analysis 

Genomic DNA was extracted from an amniotic fluid specimen using standard protocols. SOMA was performed using the Affymetrix Cytoscan HD array (Thermo Fisher Scientific Inc.—Life Technologies, Waltham, MA, USA), according to the manufacturer’s instructions. Results were analyzed using the GeneChip Command Console software and the Chromosome Analysis Suite (ChAS version 3.3) from Affymetrix (Thermo Fisher Scientific Inc.—Life Technologies, Waltham, MA, USA) with the hg19/GRCh37 human genome build. Losses and gains were considered for analysis with a minimum number of 25 probes as a reliable indicator for loss and 50 probes for gain. The criteria for a reportable copy number result in a prenatal setting include: (1) DNA copy loss or gain ≥ 100 kb within the targeted panel of clinically significant gene regions. (2) DNA copy loss or gain outside the targeted regions with a contiguous dosage change across an interval of 1000 kb or greater (for both gain and deletions), a density of 20 or greater probes per 100 kb segment, and at least one OMIM disease annotated gene. The criteria for a reportable copy number result in a postnatal setting include: (1) DNA copy loss or gain ≥ 50 kb within the targeted panel of clinically significant gene regions. (2) DNA copy loss or gain outside the targeted regions with a contiguous dosage change across an interval of 200 kb or greater (for deletions) and 500 kb or greater (for gains/duplications), a density of 20 or greater probes per 100 kb segment, and at least one OMIM disease annotated gene. Copy number variants (CNV) were evaluated for pathogenicity following ACMG and ClinGen guidelines [6].

### 2.3. Whole Exome Sequencing (WES)

For the WES testing, DNA was extracted from buccal specimens (trio WES). The exome sequencing library was prepared from genomic DNA using the KAPA Hyper Prep kit (Roche, Basel, Switzerland, KK8504). The amplified dsDNA products were further processed for capture-based library enrichment using IDT’s xGen Exome Research, CNV backbone, Human mtDNA research, and Human ID Research Panel probes, along with the xGen Hybridization and Wash kit. Paired-end sequencing was performed on the Illumina NovaSeq 6000 sequencing platform (Illumina, San Diego, CA, USA). The sequence data were aligned to the Human Reference Genome (hg19/GRCh37), and variant calls were made using DRAGEN v3.8.4 (Illumina, San Diego, CA, USA). Variant filtering and annotation were performed using an in-house pipeline developed by the Precision Genomics Laboratory at Columbia University. Variants were assessed for pathogenicity following ACMG standards and guidelines [7]. 

## 3. Results

Here, we describe a 28-month-old female who is the product of a non-consanguineous union between a mother of Irish/English/Italian descent and a father of Ukrainian/Irish/Italian descent. The family history is notable for speech delay (two paternal first cousins and the maternal grandfather). The patient’s mother was 33 years old with a primary medical history of obesity (BMI~60), asthma, and infertility. The patient was conceived via in vitro fertilization (IVF) due to maternal polycystic ovary syndrome. The embryo had preimplantation genetic testing for aneuploidies (PGT-A) with normal results. Prenatally, the patient was diagnosed with myelomeningocele, Chiari II malformation, and bilateral ventriculomegaly based on fetal imaging studies. Chromosomal and SOMA analyses were performed prenatally in our institution on the patient with amniotic fluid but did not reveal notable findings.

The patient was born at term (37w0d) via C-section after a pregnancy that was complicated by gestational diabetes, placenta previa, and fetal anomalies. Birth weight was 2.4 kg (3rd centile, Z = −1.91), with a length of 46.5 cm (11th centile, Z = −1.21) and a head circumference of 33.5 cm (22nd centile, Z = −0.78). She spent three weeks in the NICU for management of prenatally diagnosed conditions, and her surgery histories included myelomeningocele repair, ventriculomegaly VP shunt placement, and CSF shunt. Additional clinical manifestations of the patient include an umbilical hernia, a neurogenic bladder, and congenital hydronephrosis. She has been followed by neurology, neurosurgery, urology (for neurogenic bladder), GI (for constipation), and rehabilitation medicine. Brain MRI at 14 months of age revealed subependymal gray matter heterotopia, callosal dysgenesis with hypoplastic genu/rostrum, and absent splenium. Upon evaluation by neurology at 20 months of age, she was noted to be microcephalic with around 50 individual words, some scripting, and echolalia. She has been receiving physical therapy, occupational therapy, and speech-language therapy through early intervention. On an exam at 25 months of age by neurology, her height was 84.8 cm (25th centile, Z = −0.69), with a weight of 12.5 kg (54th centile, Z = 0.11) and a head circumference of 48.3 cm (66th centile, Z = 0.41). She was noted to have fleeting eye contact and poor joint attention, and she continued to have hand stereotypies. No regression in any development domain was observed. During the orthopedic surgery visit when she was 25 months old, her congenital positional left clubfoot appeared to be stable, and her right foot calcaneovalgus deformity was found to have significantly improved with conservative management of both feet. A genetic workup was recommended given her brain abnormalities.

**Figure 1:**
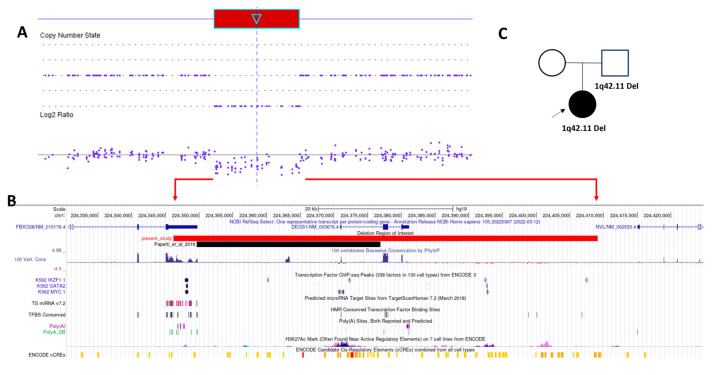
(**A**) Copy number state and Log2 ratio graphics of SOMA in the patient show a 63 kb interstitial deletion in chromosome 1q42.11 (chr1:224346184_224409144). (**B**) UCSC browser view of the deleted region detected in our study and the study by Papetti et al. [8] with tracks showing evolution conservation elements, transcription factor binding sites, predicted microRNA target sites, reported and predicted Poly(A) sites, histone modification marks, and cis-regulatory elements. (**C**) The pedigree of the study family. SOMA showed the 63-kb deletion was paternally inherited.

A trio of WES was performed postnatally but did not identify significant variants, including variants of uncertain significance, relevant to the phenotypes. In parallel, the prenatal SOMA result was reanalyzed with the postnatal analysis and reporting criteria. Interestingly, this reanalysis identified a 63 kb interstitial deletion in the long arm of chromosome 1, corresponding to genomic coordinates 224,346,184–224,409,144 [GRCh37/hg19] (Figure 1). The deleted region contains the entire gene of *DEGS1* and the last exon of *FBXO28*. Mutations in *DEGS1* are associated with autosomal recessive hypomyelinating leukodystrophy-18 (OMIM#: 618404), while mutations in *FBXO28* are associated with autosomal dominant developmental and epileptic encephalopathy-100 (OMIM#: 619777). However, the deleted region only includes the 3′ UTR of the *FBXO28* gene (NM_015176.4), and it is unclear how this deletion affects the gene function. To the best of our knowledge, the identical deletion seen in the proband has not been reported in the literature. This deleted region is not commonly seen in the online normal human genetic variation database which indicates a potential pathogenic role of genomic imbalance in this region. Therefore, the significance of this loss and its relationship to the phenotype of this patient is uncertain currently, and we reported it as a variant of uncertain significance according to the ACMG/CLINGEN guidelines for CNV classification [6].

To further evaluate the clinical relevance of this CNV, parental SOMA studies were recommended to determine whether this alteration represents a familial variant or a de novo change. Parental SOMA analysis revealed that the CNV is paternally inherited. Of note, the patient’s father does not have related symptoms and recently had a brain MRI due to migraines. The brain MRI did not show abnormal findings. 

## 4. Discussion

In the current study, we detected a 63 kb deletion at chromosome 1q42.11 in a 28-month-old girl presenting with brain abnormalities and developmental delay through reanalysis of prenatal SOMA results. The deleted region in our patient contains the 3′ UTR of *FBXO28* and the entire gene of *DEGS1*. The fact that no significant variants relevant to the indication were identified by trio whole exome sequencing analysis in our case further helped rule out the possibility of variants in other neurodevelopmental disorder-associated genes contributing to the phenotype. 

Previous studies of 1q41q42 microdeletion syndrome aiming at mapping the smallest region of overlap for this syndrome identified a ~120 kb region that contained a single gene, *FBXO28*, resulting in its proposed critical role in contributing to the syndrome [3]. Later, reports of patients with parallel clinical manifestations and smaller deletions involving *FBXO28* further pointed to a role for *FBXO28* in the 1q41q42 microdeletion syndrome, particularly in the context of neurodevelopmental and epileptic phenotypes [5,8]. 

More recently, studies by Balak et al. and Schneider et al. implicated *FBXO28* in causing a monogenic disease recognized as autosomal dominant developmental and epileptic encephalopathy 100 (OMIM#: 619777). To date, 10 cases with pathogenic *FBXO28* variants (missense, nonsense, and frameshift variants) have been described. Common clinical features include seizures, intellectual disability, and variable brain abnormalities, which are shared findings in the 1q41q42 microdeletion syndrome [2,4]. 

The neurodevelopmental phenotype (i.e., developmental delay and brain imaging abnormalities) observed in our patient aligns closely with reported cases of 1q41q42 deletion encompassing *FBXO28* and observations associated with pathogenic *FBXO28* coding variants (Table 1). Seizures are present in over 75% of patients 2 years old or younger who harbor deletions in an ~300 kb critical region containing *FBXO28*, and all patients carrying pathogenic *FBXO28* sequence variants experience seizures, with age of onset ranging from 8 months to 5 years [2,4]. Given the age of our patient and the relatively broad range of seizure onset ages, it is likely worthwhile to continue monitoring the patient for this phenotype.

The deletion detected in our patient involves the 3′ UTR of *FBXO28* without changing the coding sequence. Interestingly, a case with a 27 kb deletion involving the distal portion of the *FBXO28* 3′ UTR has been described previously [8]. The patient was noted to exhibit developmental delay, speech impairment, motor stereotypes, and seizures, in addition to abnormal brain MRI findings (Table 1). The 3′ UTR often contains regulatory regions that influence gene expression post-transcriptionally by regulating mRNA-based processes, such as mRNA stability, localization, and translation efficiency. Additionally, 3′ UTR-mediated protein-protein interactions have been shown to regulate protein posttranslational modifications, complex formation, and protein conformational changes [9]. Like the above-mentioned report by Papetti et al., studies have repeatedly underscored the pathophysiologic relevance of the 3′ UTR in neurodevelopmental disorders [10], including the Var321 mutation in *SLITRK1* associated with Tourette’s syndrome [11]. Indeed, in a recent study by Chaudhuri et al., 3′ UTR single nucleotide variants in *FBXO28* were identified in mesial temporal lobe epilepsy patients, and structural modeling of variant 3′ UTR segments revealed altered secondary and tertiary structures that could modulate mRNA stability and protein and microRNA binding, thereby potentially impacting protein abundance [12]. Adding to the concept of functionally important *FBXO28* 3′ UTR is the presence of evolutionarily conserved elements and regulatory elements (i.e., transcription factor binding sites, microRNA binding sites, cis-regulatory elements, and histone modification marks) in this region (Figure 1C). Future studies, such as RNA sequencing, may be valuable in assessing the functional importance of the *FBXO28* 3′ UTR. 

In the Papetti et al. study, the deletion involving the distal portion of the *FBXO28* 3′ UTR and exon 1 of *DEGS1* (Figure 1B) was also present in the proband’s father, who was cognitively normal without a personal history of neurological disorders and dysmorphic features [8]. Similarly, the deletion in our patient was found to have been inherited from her father, who appears to be healthy without neurological disorders. In contrast, all but two identified *FBXO28* coding variants were de novo. These two patients inherited the variants from clinically unaffected parents who were mosaic for the variant (Table 1). Together, these observations raise intriguing hypotheses and questions that need to be addressed. First, it is possible that the loss of *FBXO28* 3′ UTR is associated with incomplete phenotypic penetrance. Clinical penetrance is in part a function of the variants in question. While relatively few studies have focused on low-penetrance variants to identify molecular features responsible for their low penetrance, reduced penetrance associated with some variant types and locations in the gene/protein is not infrequently encountered in inherited diseases [13,14]. Notably, all but two of the identified pathogenic sequence variants in FBXO28 encephalopathy are located close to each other in the final exon of *FBXO28*. This led to the hypothesis of a disease mechanism that is different from the proposed haploinsufficiency in 1q41q42 microdeletion syndrome [2,4]. Given our current understanding of *FBXO28* and its associated diseases, as well as a major predicted *FBXO28* 3′ UTR functional impact thus far (i.e., modulation of gene expression and protein abundance) [12], it is probably not surprising if *FBXO28* variants impacting its 3′ UTR but not the coding sequence are associated with reduced penetrance. As exemplified by variants in untranslated *RPSA* exons with predicted functional impact comparable to fully penetrant variants but incomplete penetrance in the context of isolated congenital asplenia, the phenotypic penetrance may be modulated by various additional mechanisms [15]. Likewise, the lack of apparent phenotype in the proband’s father may also be explained by the action of yet-to-be-identified cis- and trans-acting genetic modifiers, epigenetic changes, somatic mosaicism, sex difference, and environmental factors, among others [14]. More detailed future studies of the extended family may help provide insights into these possibilities. 

Furthermore, the deletions detected in our patient and the Papetti et al. study also involve the *DEGS1* gene. While existing evidence points to *FBXO28* as an important potential contributor to the phenotype, we cannot rule out the possibility of *DEGS1*′s relation to the patient’s phenotype. *DEGS1* is widely expressed across tissues, including in the brain, and it encodes a member of the membrane fatty acid desaturase family that catalyzes the dihydroceramide to ceramide conversion in the final step of the de novo ceramide biosynthetic pathway [16]. Homozygous or compound heterozygous variants in *DEGS1* have been associated with hypomyelinating leukodystrophy-18 (OMIM#:618404), an autosomal recessive neurologic disorder characterized by the onset of global developmental delay typically in early infancy [17]. Since dual modes of inheritance have been observed in association with neurodevelopmental disorders of varying severity [18,19], the possibility of *DEGS1* contributing to the patient’s phenotype remains viable and requires further investigation.

Our proband presents with multiple congenital anomalies, and the pregnancy was complicated by fetal anomalies and maternal conditions, including obesity, polycystic ovary syndrome, infertility requiring assisted reproductive technology, and gestational diabetes. Since a myriad of maternal factors are known to influence the health of the child during pregnancy and long after birth, the complex clinical manifestations of the patient could also be attributed, at least partly, to these other complicating risk factors [20,21,22,23].

Chromosomal microarray analysis has been recommended by the American College of Obstetricians and Gynecologists and the Society for Maternal-Fetal Medicine as the initial genetic test of choice for fetuses with ultrasound anomalies and stillbirth [24]. Reporting of variants of uncertain significance, especially in the prenatal setting, remains a topic for active discussion due to concerns about their consequences, including psycho-social considerations [25]. The copy number change in our patient was reported after reanalysis of prenatal SOMA results with postnatal whole genome setting and reporting criteria when the knowledge base had also evolved, which speaks to the differences in CNV analysis and reporting between prenatal and postnatal settings and the benefits of data reanalysis when clinically indicated.

In conclusion, we report a novel copy number loss at chromosome 1q42 that was identified through postnatal reanalysis of prenatal SOMA results, emphasizing the different goals and reporting criteria between prenatal and postnatal tests. Our study provides evidence supporting the hypothesis that *FBXO28* is a gene contributing to the chromosome 1q41q42 deletion syndrome, and it also highlights the potential functional importance of the *FBXO28* 3′ UTR region. Further investigations are still needed to evaluate the clinical relevance of this 63 kb deletion in relation to the proband’s phenotypes.

## Figures and Tables

**Table 1:** Clinical features of individuals with *FBXO28* 3′ UTR deletions and coding region variants.

Reference	Present Study	Papetti et al. [8]	Balak et al. [2]; Schneider et al. [4]
**Gender**	1 female	1 female	6 female:4 male
**Age at report (range)**	28 months	5 years	11 months–25 years
**Variant**	Deletion including *DEGS1* and 3′ UTR of *FBXO28*	Deletion including exon 1 of *DEGS1* and distal portion of the *FBXO28* 3′ UTR	Variants of *FBXO28* coding regions (missense, nonsense, and frameshift)
**Coordinates (GRCh37/hg19)**	chr1: 224,346,184–224,409,144	chr1: 224,349,658–224,376,898	NA
**Inheritance**	Paternally inherited	Paternally inherited	De novo (8/10); Inherited from a mosaic parent (2/10)
**Brain abnormalities**	Chiari II malformation, bilateral ventriculomegaly, subependymal gray matter heterotopia, callosal dysgenesis with hypoplastic genu/rostrum, and absent splenium	Chiari type I malformation	Atrophy; delayed/abnormal myelination; simplified gyral pattern; pachygyria; cortical dysplasia; polymicrogyria (9/10)
**Intellectual disability/developmental delay**	Yes	Yes	Yes (9/10)
**Movement disorder/stereotypies/repetitive behaviors**	Hand stereotypies	Motor stereotypies with repetitive hands and head movements; behavioral disturbance	Myoclonus; stereotypies; hyperkinetic movements; choreoathetosis; ataxia; dystonia; dyskinesia (8/10)
**Dysmorphic features**	Microcephaly	Low-set ears and hypertelorism	Acquired microcephaly; high palate; short palpebral fissures; hypertelorism; broad and flat/depressed nasal bridge/root; full lips; gingival hyperplasia; micrognathia (6/10)
**Hypotonia**	No	NA	Yes (6/10)
**Seizure**	No	Yes (episodes of generalized febrile seizures started between 18 mo–3 y)	Yes (11/11; age of onset 8 mo–5 y)
**Other anomalies**	Myelomeningocele, umbilical hernia, congenital hydronephrosis; left clubfoot deformity and right calcaneovalgus foot	No	Contractures (3/10); skeletal abnormalities (3/10); ophthalmologic abnormalities (2/10)

NA = not available.

## Data Availability

All the data that supports the findings of this study are included in this published article.

## References

[1] Zhang F., Gu W., Hurles M.E., Lupski J.R. (2009). Copy number variation in human health, disease, and evolution. Annu. Rev. Genom. Hum. Genet..

[2] Balak C., Belnap N., Ramsey K., Joss S., Devriendt K., Naymik M., Jepsen W., Siniard A.L., Szelinger S., Parker M.E. (2018). A novel FBXO28 frameshift mutation in a child with developmental delay, dysmorphic features, and intractable epilepsy: A second gene that may contribute to the 1q41-q42 deletion phenotype. Am. J. Med. Genet. Part A.

[3] Au P.B., Argiropoulos B., Parboosingh J.S., Innes A.M. (2014). Refinement of the critical region of 1q41q42 microdeletion syndrome identifies FBXO28 as a candidate causative gene for intellectual disability and seizures. Am. J. Med. Genet. Part A.

[4] Schneider A.L., Myers C.T., Muir A.M., Calvert S., Basinger A., Perry M.S., Rodan L., Helbig K.L., Chambers C., Gorman K.M. (2021). FBXO28 causes developmental and epileptic encephalopathy with profound intellectual disability. Epilepsia.

[5] Cassina M., Rigon C., Casarin A., Vicenzi V., Salviati L., Clementi M. (2015). FBXO28 is a critical gene of the 1q41q42 microdeletion syndrome. Am. J. Med. Genet. Part A.

[6] Riggs E.R., Andersen E.F., Cherry A.M., Kantarci S., Kearney H., Patel A., Raca G., Ritter D.I., South S.T., Thorland E.C. (2020). Technical standards for the interpretation and reporting of constitutional copy-number variants: A joint consensus recommendation of the American College of Medical Genetics and Genomics (ACMG) and the Clinical Genome Resource (ClinGen). Genet. Med..

[7] Richards S., Aziz N., Bale S., Bick D., Das S., Gastier-Foster J., Grody W.W., Hegde M., Lyon E., Spector E. (2015). Standards and guidelines for the interpretation of sequence variants: A joint consensus recommendation of the American College of Medical Genetics and Genomics and the Association for Molecular Pathology. Genet. Med..

[8] Papetti L., Schettini L., Garone G., Gennaro E., Malacarne M., Properzi E., Spalice A. (2016). The crucial role of FBXO28 in the pathogenesis of the 1q41q42 microdeletion syndrome. Am. J. Med. Genet. Part A.

[9] Mayr C. (2019). What Are 3′ UTRs Doing?. Cold Spring Harb. Perspect. Biol..

[10] Wanke K.A., Devanna P., Vernes S.C. (2018). Understanding Neurodevelopmental Disorders: The Promise of Regulatory Variation in the 3′UTRome. Biol. Psychiatry.

[11] Abelson J.F., Kwan K.Y., O’Roak B.J., Baek D.Y., Stillman A.A., Morgan T.M., Mathews C.A., Pauls D.L., Rasin M.-R., Gunel M. (2005). Sequence variants in SLITRK1 are associated with Tourette’s syndrome. Science.

[12] Chaudhuri T., Chintalapati J., Hosur M.V. (2021). Identification of 3′-UTR single nucleotide variants and prediction of select protein imbalance in mesial temporal lobe epilepsy patients. PLoS ONE.

[13] Cooper D.N., Krawczak M., Polychronakos C., Tyler-Smith C., Kehrer-Sawatzki H. (2013). Where genotype is not predictive of phenotype: Towards an understanding of the molecular basis of reduced penetrance in human inherited disease. Hum. Genet..

[14] Kingdom R., Wright C.F. (2022). Incomplete Penetrance and Variable Expressivity: From Clinical Studies to Population Cohorts. Front. Genet..

[15] Bolze A., Boisson B., Bosch B., Antipenko A., Bouaziz M., Sackstein P., Chaker-Margot M., Barlogis V., Briggs T., Colino E. (2018). Incomplete penetrance for isolated congenital asplenia in humans with mutations in translated and untranslated RPSA exons. Proc. Natl. Acad. Sci. USA.

[16] Chaurasia B., Tippetts T.S., Mayoral Monibas R., Liu J., Li Y., Wang L., Wilkerson J.L., Sweeney C.R., Pereira R.F., Sumida D.H. (2019). Targeting a ceramide double bond improves insulin resistance and hepatic steatosis. Science.

[17] Pant D.C., Dorboz I., Schluter A., Fourcade S., Launay N., Joya J., Aguilera-Albesa S., Yoldi M.E., Casasnovas C., Willis M.J. (2019). Loss of the sphingolipid desaturase DEGS1 causes hypomyelinating leukodystrophy. J. Clin. Investig..

[18] McNeill A., Iovino E., Mansard L., Vache C., Baux D., Bedoukian E., Cox H., Dean J., Goudie D., Kumar A. (2020). SLC12A2 variants cause a neurodevelopmental disorder or cochleovestibular defect. Brain.

[19] McNeill A. (2021). Comment on: Bi-allelic variants in genes previously associated with dominant inheritance: CACNA1A, RET and SLC20A2. Eur. J. Hum. Genet..

[20] Muglia L.J., Benhalima K., Tong S., Ozanne S. (2022). Maternal factors during pregnancy influencing maternal, fetal, and childhood outcomes. BMC Med..

[21] Chen S., Fan M., Lee B.K., Dalman C., Karlsson H., Gardner R.M. (2023). Rates of maternal weight gain over the course of pregnancy and offspring risk of neurodevelopmental disorders. BMC Med..

[22] Kwok J., Speyer L.G., Soursou G., Murray A.L., Fanti K.A., Auyeung B. (2023). Maternal metabolic syndrome in pregnancy and child development at age 5: Exploring mediating mechanisms using cord blood markers. BMC Med..

[23] Chen S., Persson M., Wang R., Dalman C., Lee B.K., Karlsson H., Gardner R.M. (2023). Random capillary glucose levels throughout pregnancy, obstetric and neonatal outcomes, and long-term neurodevelopmental conditions in children: A group-based trajectory analysis. BMC Med..

[24] Committee on Genetics and the Society for Maternal-Fetal Medicine (2016). Committee Opinion No.682: Microarrays and Next-Generation Sequencing Technology: The Use of Advanced Genetic Diagnostic Tools in Obstetrics and Gynecology. Obstet. Gynecol..

[25] Watts G., Newson A.J. (2021). To offer or request? Disclosing variants of uncertain significance in prenatal testing. Bioethics.

